# Sequential storage and release of microdroplets

**DOI:** 10.1038/s41378-021-00303-9

**Published:** 2021-09-29

**Authors:** Zenon Toprakcioglu, Tuomas P. J. Knowles

**Affiliations:** 1 Centre for Misfolding Diseases, Yusuf Hamied Department of Chemistry, University of Cambridge, Lensfield Road, Cambridge, CB2 1EW UK; 2 Cavendish Laboratory, Department of Physics, University of Cambridge, J J Thomson Avenue, Cambridge, CB3 0HE UK

**Keywords:** Chemistry, Engineering

## Abstract

Droplet microfluidic methods have opened up the possibility of studying a plethora of phenomena ranging from biological to physical or chemical processes at ultra low volumes and high throughput. A key component of such approaches is the ability to trap droplets for observation, and many device architectures for achieving this objective have been developed. A challenge with such approaches is, however, recovering the droplets following their confinement for applications involving further analysis. Here, we present a device capable of generating, confining and releasing microdroplets in a sequential manner. Through a combination of experimental and computational simulations, we shed light on the key features required for successful droplet storage and retrieval. Moreover, we explore the effect of the flow rate of the continuous phase on droplet release, determining that a critical rate is needed to ensure complete droplet deformation through constrictions holding the droplets in place prior to release. Finally, we find that once released, droplets can be retrieved and collected off chip. The ability to generate, store and sequentially release droplets renders such a device particularly promising for future applications where reactions may not only be monitored on-chip, but droplets can also be retrieved for further analysis, facilitating new exploratory avenues in the fields of analytical chemistry and biology.

## Introduction

Microfluidic technologies are increasingly being used for a variety of applications involving the observation of chemical and biochemical processes on small scales^1–4^. Due to their high throughput, such techniques are well suited for studying enzymatic^5^ and single-cell assays^6,7^, probing PCR reactions^8^, monitoring phase transition phenomena such as protein aggregation^9–14^ and have also been used for sizing and separating molecular species in complex solutions^15^. More specifically droplet-microfluidics, where two immiscible phases intersect resulting in the formation of micrometer-sized droplets^1,2,16,17^, has emerged as a powerful technique for probing reactions since each droplet forms a microcomponent and effectively acts as an individual microreactor^18–20^. Such water-in-oil droplets are typically formed using flow-focusing devices, which have allowed for the generation of monodisperse droplets that can be precisely controlled by varying the dispersed and continuous phase flow rates^21,22^. Moreover, droplet-microfluidics has provided an avenue for analysing, manipulating and sorting droplets through techniques such as FACS (fluorescence activated cell sorting)^23–25^.

In order to monitor processes occurring within droplets, array-based technologies capable of confining microdroplets have emerged^26^. Such approaches include incorporating holes or wells into the device design or by introducing electrical fields to isolate droplets^27–32^. Alternative ways of confining droplets can be achieved through the use of valves; however, these trapping devices are quite involved and usually require a specialist to operate^33,34^. More recently, multi-layered lithography has been employed to produce three-dimensional microfluidic devices capable of trapping cells for studying them on-chip^35,36^. Although there are multiple methods of confining microdroplets, there are very few, if any, devices capable of both storing and retrieving droplets. Such a tool would allow for both the parallel study of events occurring within droplets but also subsequent further analysis of the droplets following retrieval. This would be extremely useful for cell-related assays and applications^37,38^, where cells could be encapsulated within droplets, stored on-chip to monitor a particular reaction, retrieved and then sorted.

Here, we present a device architecture capable of successfully storing and releasing microdroplets in a sequential manner. Due to the device geometry, droplets are stored in single occupancy per well, and are then retrieved in sequence, making such a device suitable for systematically monitoring reactions within microdroplets, followed by droplet collection for further analysis. We show through simulation and experimental data that device architecture is instrumental in defining the success of droplet release. Furthermore, we determined that for a particular constriction size, there is a critical flow velocity below which droplets cannot be released. Finally, we found that following release, droplets may be retrieved and sequentially collected making this device ideal for applications where both on-chip storage and further analysis is vital.

## Results and discussion

### Effect of constriction size and flow rate on droplet release

**Fig. 1:**
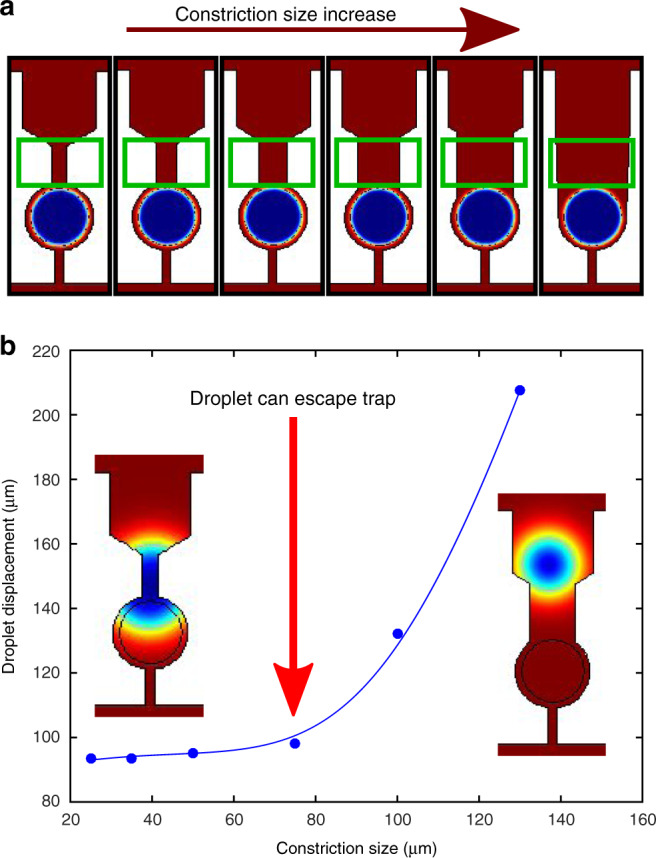
Simulation results showing the effect of constriction size on droplet release. **a** Finite elements simulation results showing the effect of a gradual increase of the primary constriction size ranging from 35 μm (left panel) to 130 μm (right panel). The aqueous droplet (blue) is set at the center of the trapping chamber, surrounded by the continuous oil phase (red). The constriction area is denoted by a green box. **b** Graph of droplet displacement as a function of constriction size based on simulation results. The velocity used for all simulations was 0.03 ms^−1^.

In order to confine and subsequently release droplets, a device geometry consisting of a droplet-maker and an array of confinement chambers was designed. The trapping array architecture consists of two compartments. The first compartment guides the droplet through the primary constriction into the second compartment, which in turn has a constriction which is too narrow for the droplet to deform through and thus confinement is achieved (Fig. 1a). The process of droplet trapping is shown in Supplementary Information video 1, while and image showing trapped droplets is shown in Fig. S1. Once trapped, by flowing the continuous phase from the outlet, droplet release can be achieved. This is demonstrated in the time-lapse video showing droplet release and sequential retrieval within the device (Supplementary Information video 2). Therefore, such a device is ideal for the study of monitoring reactions within droplets by confinement and subsequently retrieving the droplets sequentially for further analysis or for sorting.

In order to probe the effect of primary constriction size on the ability of the droplet to escape the trap, finite element simulations were conducted using COMSOL software. A two phase laminar flow model was employed, where water and oil were considered as the two immiscible fluids. The device geometry was initially imported onto COMSOL software, while typical viscosity and interfacial tension values of deionised water and fluorinated oil were used.

Six different constriction sizes were considered; 25, 35, 50, 75, 105 and 130 μm. For each simulation conducted, the velocity of the continuous (oil) phase was set at 0.03 ms^−1^, while each simulation was run until completion, i.e. until an equilibrium was reached and the aqueous droplet stopped moving through the microfluidic channels. The constriction sizes were systematically varied (Fig. 1a), and the droplet displacement as a function of constriction size is plotted in Fig. 1b. The distance that the droplet travelled was determined by measuring the interval from the center of the microdroplet to the final “resting” point reached. It is clear from Fig. 1b that initially, droplet movement does not significantly vary for smaller constriction sizes. However, at 75 μm, the flow rate is high enough to allow droplet deformation through the primary constriction thus allowing for a successful escape event, i.e. there is a threshold beyond which droplets can be released. This behaviour increases sharply for larger constriction sizes until there is essentially no constriction (right panel in Fig. 1a).

**Fig. 2:**
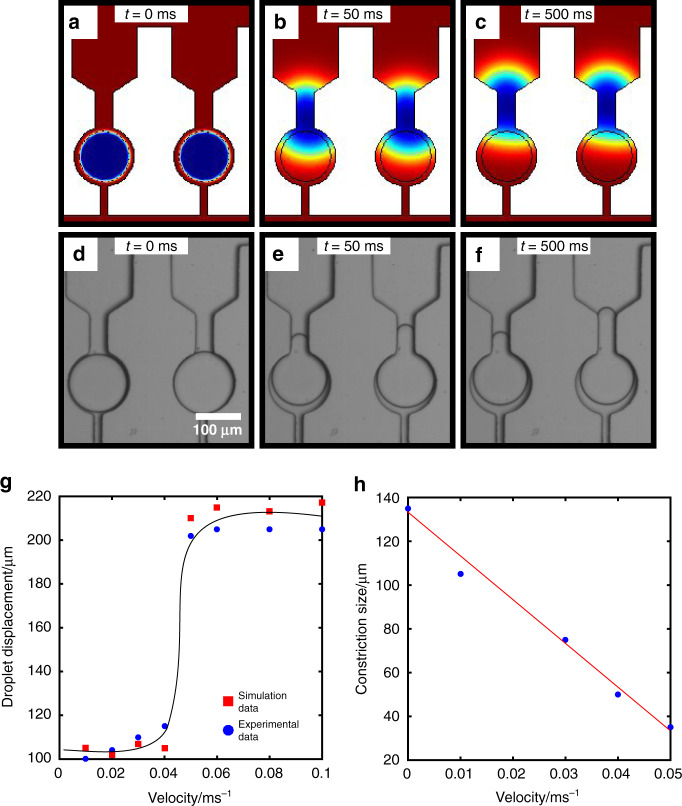
Simulation and experimental results showing the effect of flow rate on droplet release. **a**–**c** Finite elements simulation results of partial droplet deformation through the primary constriction enabled by insufficient velocity in the continuous phase (red). **a** The droplets (blue) are initially in the traps. **b** They then partially deform through the constriction. **c** After 500 ms of simulation time the droplets still remain in this partial deformed state. **d**–**f** Time evolution bright field image sequence of trapped droplets showing partial deformation through the primary constriction. **d** Droplets trapped in the chambers. **e, f** Partial deformation of droplets through the primary constriction. After 500 ms the droplets still remain in partially deformed through the constriction. **g** Graph of droplet movement as a function of velocity for a constriction size of 35 μm. Red squares correspond to simulation results whereas blue points correspond to experimental data. **h** Graph of constriction size against velocity needed for a droplet to escape.

The dependence of the continuous phase velocity on droplet movement through the primary constriction was next investigated. We initially explored in close detail how an insufficient flow rate allows for partial droplet deformation through a constriction size, but does not allow for complete droplet release (Fig. 2a–f). A constriction size of 35 μm was chosen and both simulations and experimental data were obtained in order to investigate this. The velocity of the continuous phase for both simulation and experiments was set at 0.03 ms^−1^. As can be seen in the simulation results in Fig. 2a–c, droplets are initially deformed through the constriction after 50 ms of simulation time (Fig. 2b). They still remain in this configuration even after 500 ms of simulation time, as the flow rate is not high enough to push them completely through the constriction (Fig. 2c). This is also corroborated experimentally using bright field microscopy (Fig. 2d–f), where for the same timescales, the flow rate of the continuous phase is insufficiently high enough to push the droplet through the constriction (Fig. 2e–f). This can also be seen in the Supplementary Information video 3.

The effect of flow rate, for a given geometry, on droplet deformation and subsequent escape was then investigated. The velocity was systematically changed at a particular constriction size, and simulations were run in order to determine at which flow rate droplets can be retrieved. It is clear from the graph in Fig. 2g, which depicts droplet movement as a function of velocity for a 35 μm constriction size, that a sigmoidal behaviour is observed. Initially the effect of flow rate on droplet movement is minimal. However, there is a point beyond which a sufficient flow exists that allows droplets to successfully escape the constriction. For this particular geometry it was found to be 0.05 ms^−1^. Above this point, any velocity applied is enough to deform the droplet through the constriction and similar movements were observed. This sigmoidal behaviour was also confirmed experimentally (blue points) and similar droplet movements were found for equal continuous phase velocities. Finally, a graph of constriction size against velocity was plotted (Fig. 2h). A linear relation was established between these two variables while it was found that for larger constriction sizes, smaller velocities are needed for droplet release.

**Fig. 3:**
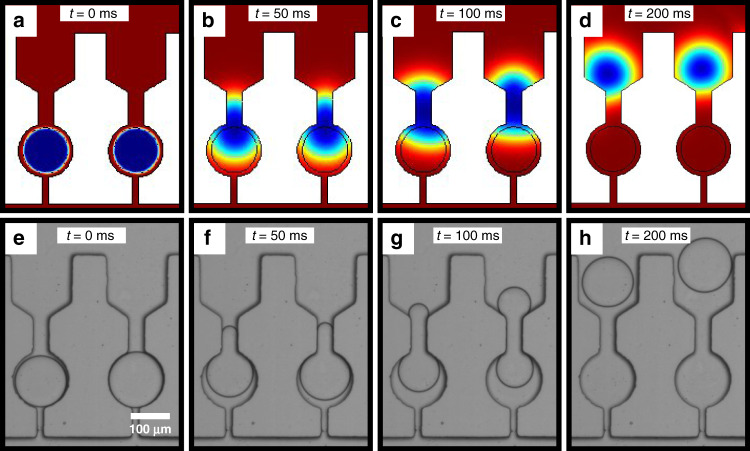
Simulation and experimental data of droplet release at different time frames. **a**–**d** Finite elements simulations showing droplet release at different timepoints. The primary constriction size was 35 μm, while the velocity used was 0.05 ms^−1^. **e**–**h** Time evolution bright field image sequence of trapped droplets showing complete deformation through the primary constriction and subsequent release.

We next sought to capture, through a combination of experiment and simulation, a droplet escape/release event. This is shown in Fig. 3, where panels a–d show simulation results at different timepoints of two trapped droplets deforming through a 35 μm-sized constriction and their reformation on the other side of the trap. This escape process is also shown experimentally in Fig. 3e–h, where a time-lapse microscopy image sequence reveals that for the same timepoints as those used for the simulations, droplet deformations/reformations take place in a highly correlated manner.

### Droplet release and retrieval

**Fig. 4:**
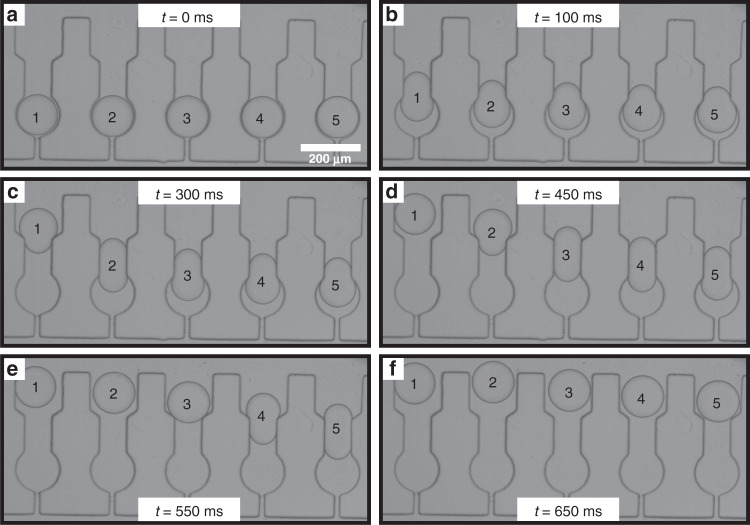
Droplet release occurring in a sequential manner. **a**–**f** Bright field time-lapse microscopy of sequential droplet release. **a** Initially at t = 0 ms, all droplets are trapped. **b** After 100 ms, droplet 1 starts moving through the constriction with the other droplets following in succession. **c**–**e** Sequentially each droplet starts moving through the constriction. **f** Finally after 650 ms all droplets have been successfully released.

Droplet release and retrieval was then studied in more detail. We initially investigated whether sequential release could be achieved. A device architecture consisting of a primary constriction of 75 μm was chosen for this study. Following both simulation and experimental results, we found that a constriction size of 75 μm allows for not only the storage of microdroplets, but also for their subsequent release, without the need to apply high flow rates. For this reason, a constriction geometry of 75 μm rather than 35 μm was used for this part of the study. By flowing the continuous oil phase at 0.03 ms^−1^, droplet release can be seen in the bright field images (Fig. 4).

Since each trap within the device is connected through a long serpentine, the fluid flow has two alternative “pathways” that it can follow. Either it can go through the full length of the serpentine channel, or it can flow through the traps, in a perpendicular direction to the serpentine channel. It is known that the fluid flow will always follow the path of least resistance, and even though the traps have narrow constrictions in comparison to the serpentine channel, their large number ensures that they present a much larger total cross-sectional area. Thus, the flow predominantly goes through the constrictions and pushes each droplet sequentially through the narrower primary constriction allowing for droplets to be released in sequence. This is demonstrated in Fig. 4 and in Supplementary Information video 2, where droplet 1 is released through the constriction before droplet 2, which in turn is released before droplet 3, and so on. This phenomenon is depicted clearly in Fig. 4d, where it is evident that after pushing for 450 ms, the five droplets are at different positions within their relative traps, and only droplet 1 has successfully been fully released.

It should be noted that for different aqueous and oil systems, the critical velocity needed to deform the droplet past the constriction and into the trapping chamber will change. This effect is due to the change in interfacial tension between different oil-water systems. The Laplace pressure needed to deform a droplet with a radius of 50 μm through the 75 μm constriction and into the trap is:

1 $${{\Delta }}P\approx \gamma /r=154\,Pa$$

where *γ* is the interfacial tension between the aqueous and the fluorinated surfactant-oil phases has been taken as 5 mN/m^39^, and *r* is the radius of curvature of the droplet.

The pressure is related to the flow rate by the Hagen-Poiseuille relation for a channel with a rectangular cross section^40,41^:

2 $$Q=\frac{{{\Delta }}P{h}^{3}w}{12\eta L}-\frac{16{{\Delta }}P{h}^{4}}{L\eta {\pi }^{5}}\mathop{\sum }\limits_{n=1,3,5,...}^{\infty }\frac{1}{{n}^{5}}\tanh \left(\frac{n\pi w}{2h}\right)$$

where *w* is the width, *h* is the height, *L* is the length of the channel and *Q* is the flow rate. For the purposes of our calculation, only the first three terms were taken as the series converges rapidly and neglecting higher-order terms introduces an error of less than 1%.

Thus, we find that the calculated flow rate needed to deform the droplet is:

3 $$Q\approx 6.3x \,1{0}^{-11}{m}^{3}{s}^{-1}\approx 230\mu Lh{r}^{-1}$$

This calculation is in good agreement with the experimentally determined critical flow rates (400–800 μL/h). This implies that for oil-water systems where the interfacial tension is greater than the one used in this study, the flow rate needed to deform the droplet through the constriction would increase.

Moreover, by applying a disperse phase flow rate (*Q*_dis_) of 100 μL/h and by varying the continuous oil phase (*Q*_con_) from 400–800 μL/h, we demonstrate that systematic control over droplet diameter could be achieved while still ensuring successful trapping. This effect can be seen in the graph of droplet size as a function of the continuous phase flow rate (Fig. S2), where it was found that successful trapping could be achieved for droplet diameters in the range of 85–120 μm.

**Fig. 5:**
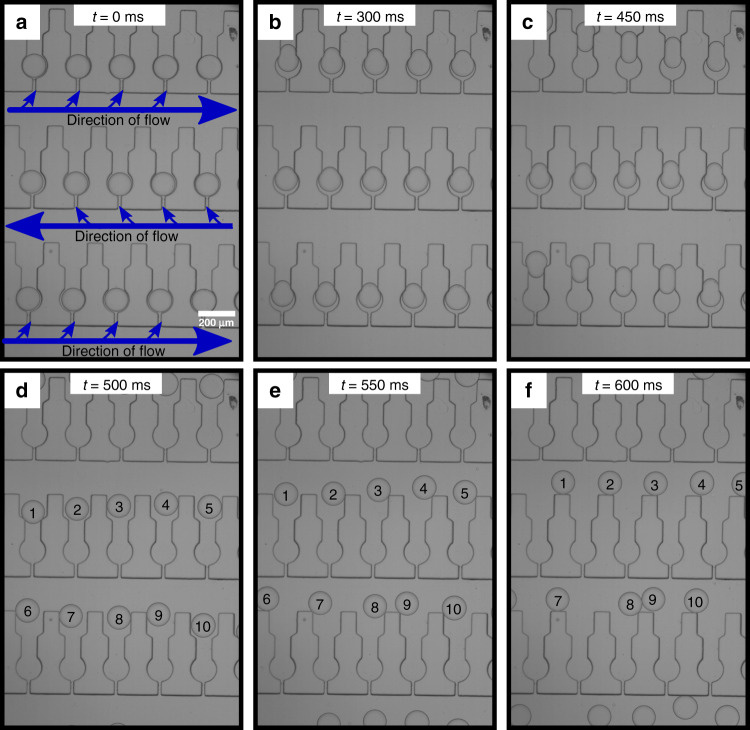
Experimental data of sequential droplet retrieval within the device. **a**–**f** Bright field time-lapse microscopy of sequential droplet retrieval. **a**–**d** Droplets escape the trap and are released. **e**–**f** The flow within the serpentine channel directs the droplet out of the device in a sequential manner.

Finally, having shown sequential droplet release, we next investigated whether retrieval, for applications involving further droplet analysis, could be achieved. As previously mentioned, the traps within the device are connected with a serpentine, so the direction of flow from one row of traps to another is in opposite directions. This is shown in Fig. 5a. After droplet release (Fig. 5b–d), all the droplets exit the guidance area and enter part of the serpentine channel. Once there, the flow directs droplets 1–5 towards the right and droplets 6–10 towards the left (Fig. 5e–f). Therefore, droplets are not only retrieved, but they are also sequentially collected as they flow through the serpentine channel in series.

**Fig. 6:**
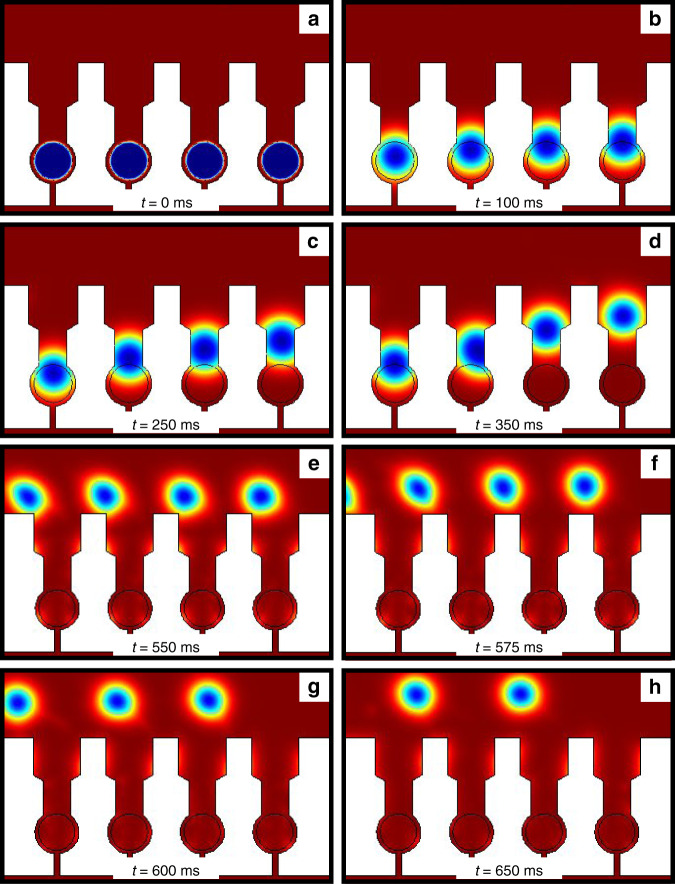
Simulation results showing droplet retrieval within the device. **a**–**d** Finite elements simulations showing sequential droplet release at different timepoints. **e**–**h** Finite elements simulations showing droplet movement and retrieval through the device.

This finding is further corroborated through simulation. These results agree with experimental data, that droplet release is achieved by a sequential manner (Fig. 6a–d). Moreover, once released, droplets can be retrieved through constant flow of the continuous phase. The droplets move opposite to their order of release because the the direction of flow in the serpentine channel varies from row to row and is in opposite directions. The simulation results in Fig. 6e–h show that for similar timescales to experimentally derived data, droplets can move through the device after they are released from the traps.

**Fig. 7:**
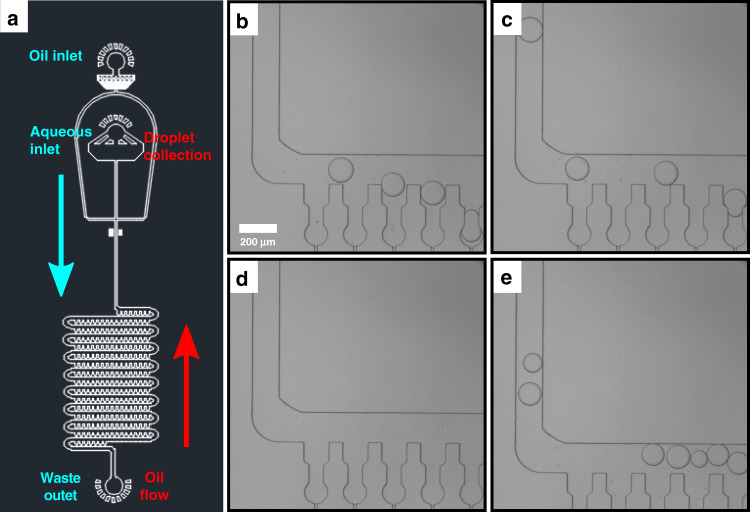
Design of the microfluidic device used to capture, store and release microdroplets. **a** Design of microfluidic device used that can store and release the microdroplets. **b**–**e** Bright field images of droplet collection following release. The scale bar for all images is 200 μm.

Droplet retrieval through the device was then investigated. The mechanism through which droplets are initially generated, trapped, released and finally retrieved can be shown in the schematic in Fig. 7a. Initially, the aqueous phase is intersected by the oil phase, resulting in the formation of water-in-oil droplets. The continuous oil phase pushes these droplets into the traps, whereby they are indefinitely confined. Oil is still continuously flowed from the oil inlet, thus ensures that no excess droplets are left in the serpentine. The tubing from the aqueous inlet is then removed and oil is subsequently pushed from the outlet. The droplets are then collected through the aqueous inlet. This process is depicted by blue arrows, which indicate fluid flow in order to trap droplets and conversely, by red arrows which show the flow during release and retrieval (Fig. 7a). Microdroplets can thus be retrieved through the serpentine and directed towards the aqueous inlet for potential further applications (Fig. 7b–e). Initially, droplets from the first row of the trapping device are collected (Fig. 7b–c). This process is followed by an interval where no droplets are observed, which corresponds to the area in-between trapping rows. The droplets from the second row are then seen to move through device (Fig. 7e) and this procedure is repeated until all the droplets are collected.

## Conclusions

In conclusion, we have designed, fabricated and demonstrated a device that is capable of sequentially storing and releasing monodisperse droplets in an array of trapping chambers. By combining simulation with experiment, we were able to probe how flow rates and constriction sizes affect droplet release. For a fixed flow rate, the effect of constriction size on effective droplet release was initially studied. Conversely, for a fixed device architecture, the velocity of the continuous phase was varied in order to monitor the behaviour of droplet release, and it was found that both experiment and computational methods give similar results. High speed imaging was used to study the partial and complete deformation of droplets as they pass through the constriction and it was found that again experiment and simulation results agree in a highly correlated manner. Finally, droplet release and retrieval was monitored and it was observed that due to the geometry of the device, sequential release of droplets through the traps takes place. Moreover, once these droplets are released, the continuous phase is able to push them through the serpentine allowing for sequential retrieval. The architecture of this device allows for the reproducible generation and storage of water-in-oil droplets and their subsequent release and retrieval. Such a device presents promising applications for encapsulating molecules within the microdroplets, trapping and storing them on-chip in order to monitor biological or chemical events and subsequently sequentially releasing and retrieving the droplets for further analysis with downstream platforms including mass-spectrometry or for single-cell sequencing.

## Materials and methods

### Device fabrication

The master used to sequentially trap and release microdroplets was fabricated using a photolithographic process. In brief, a 50 μm-thick negative photo-resist (SU-8 3050, MicroChem) was spin coated onto a silicon wafer. This was then soft baked for 25 min at 95 °C. The mask was placed onto the wafer, exposed under UV light in order to induce polymerisation and post-baked at 95 °C for 5 min^26^. Finally, in order to remove any excess photo-resist, the master was developed in Propylene glycol methyl ether acetate (PGMEA) (Sigma–Aldrich).

Microfluidic devices were fabricated using a 10:1 ratio of elastomer PDMS to curing agent (Sylgard 184, DowCorning, Midland, MI) and cured for 3 h at 65 °C. PDMS was cut, peeled off the master and holes of 0.75 mm were punched on the PDMS. The PDMS was then bonded on a glass slide after treatment with a plasma bonder (Diener Electronic, Ebhausen, Germany).

### Droplet formation

neMESYS syringe pumps (Cetoni, Korbussen, Germany) were used to control the flow rates within the channels. For water-in-oil droplets, deionised water was used as the dispersed phase, while fluorinated oil (Fluorinert FC-40, Sigma–Aldrich) containing 2% w/w fluorosurfactant (RAN biotechnologies) was used as the continuous phase. Furthermore, the use of a camera (Mikrotron High Speed Cameras) was employed to track droplets.

### Finite elements simulations

A two phase laminar flow model was used in the software package COMSOL, in order to simulate droplet release through the trap. The device architecture was imported from AutoCAD onto COMSOL prior to conducting the finite elements simulations. Literature values were used for parameters such as phase viscosities and interfacial tensions. A time-dependent model was employed in order to simulate the two phase flows.

## Supplementary information

Supplementary Information

Supplementary video

Supplementary video

Supplementary video

Supplemental Material

Supplemental Material
