# Supplementary material for: Sequential storage and release of microdroplets
Source: Microsyst Nanoeng. 2021 Sep 29;7:76. doi: 10.1038/s41378-021-00303-9 (PMC8481565; doi:10.1038/s41378-021-00303-9)
Supplement: Supplementary file 5 — Supplemental Material [file 41378_2021_303_MOESM5_ESM.pdf]

Sequential Storage and Release of Microdroplets

Zenon Toprakcioglu[†] and Tuomas P. J. Knowles[*,†,‡]

†*Centre for Misfolding Diseases, Yusuf Hamied Department of Chemistry, University of Cambridge, Lensfield Road, Cambridge CB2 1EW, UK*

‡*Cavendish Laboratory, Department of Physics, University of Cambridge, J J Thomson Avenue, Cambridge CB3 0HE, UK*

E-mail: tpjk2@cam.ac.uk

Abstract

Droplet microfluidic methods have opened up the possibility of studying a plethora of phenomena ranging from biological to physical or chemical processes at ultra low volumes and high throughput. A key component of such approaches is the ability to trap droplets for observation, and many device architectures for achieving this objective have been developed. A challenge with such approaches is, however, recovering the droplets following their confinement for applications involving further analysis. Here, we present a device capable of generating, confining and releasing microdroplets in a sequential manner. Through a combination of experimental and computational simulations, we shed light on the key features required for successful droplet storage and retrieval. Moreover, we explore the effect of the flow rate of the continuous phase on droplet release, determining that a critical rate is needed to ensure complete droplet deformation through

constrictions holding the droplets in place prior to release. Finally, we find that once released, droplets can be retrieved and collected off chip. The ability to generate, store and sequentially release droplets renders such a device particularly promising for future applications where reactions may not only be monitored on-chip, but droplets can be retrieved for further analysis, facilitating new exploratory avenues in the fields of analytical chemistry and biology.

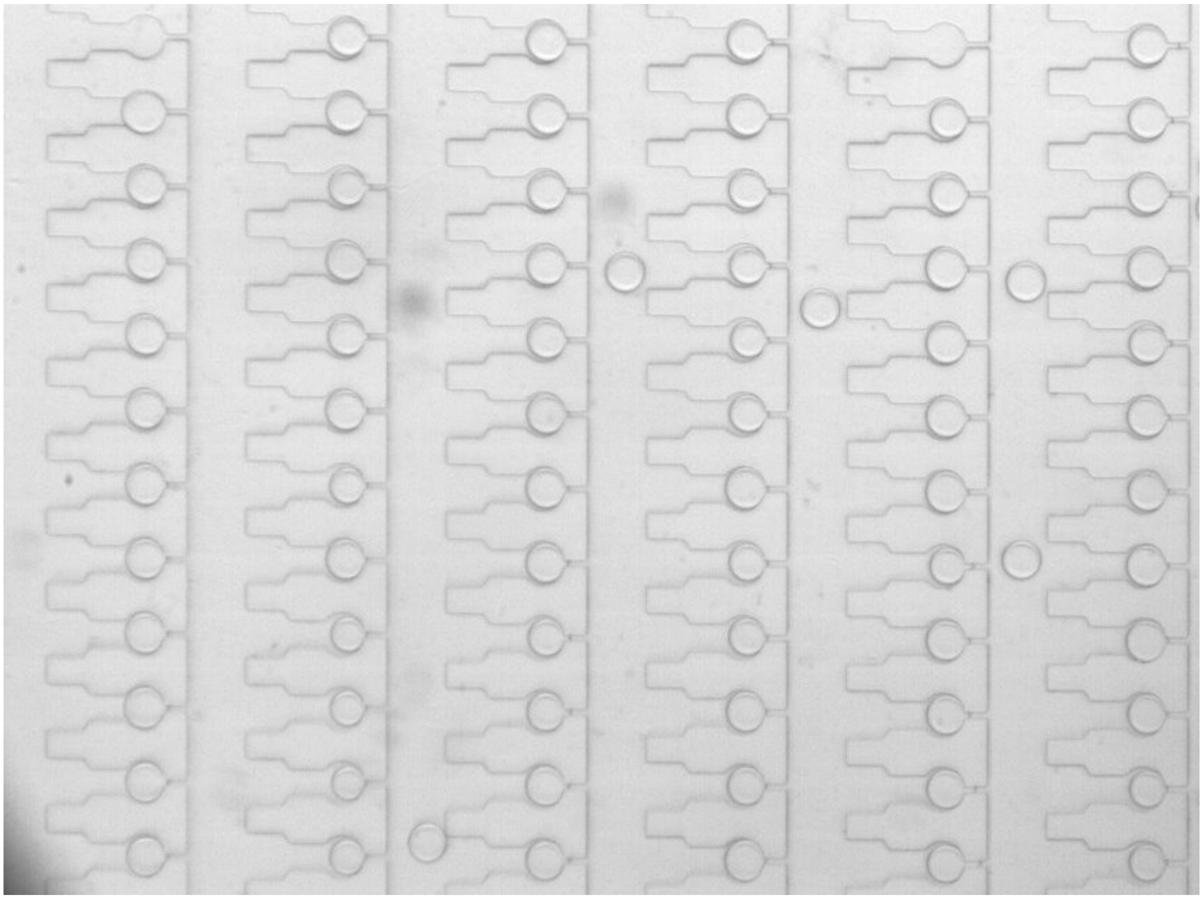

Figure S1: Bright field micrograph of droplets confined within the trapping device during continuous droplet formation.

Acknowledgments

The research leading to these results has received funding from the European Research Council under the European Union's Seventh Framework Programme (FP7/2007-2013) through the ERC grant PhysProt (agreement n° 337969). We are grateful for financial support from the BBSRC (TPJK), the Newman Foundation (TPJK), the Wellcome Trust (TPJK) and the Cambridge Centre for Misfolding Diseases.

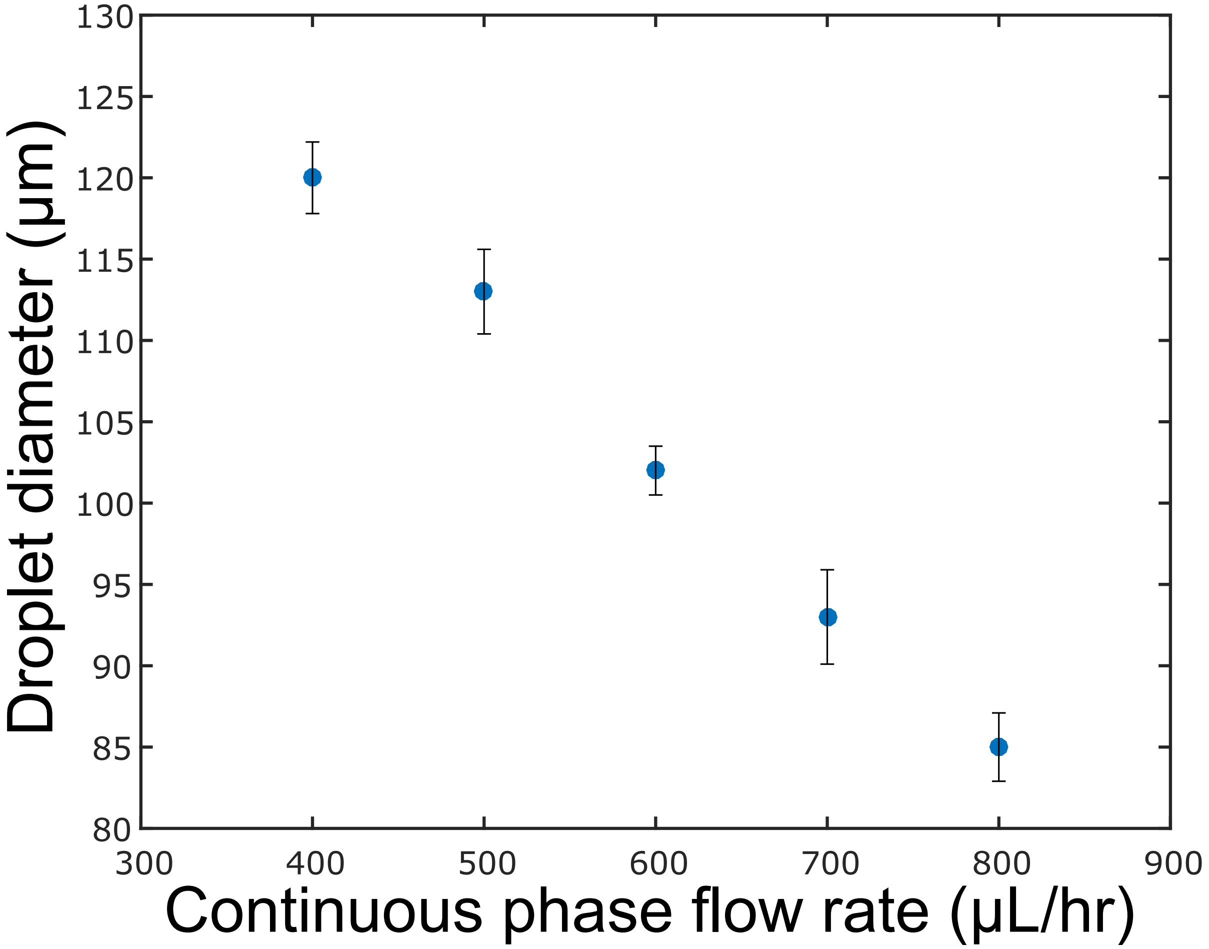

Figure S2: Graph of droplet diameter as a function of the continuous phase flow rate. The dispersed phase flow rate used for all measurements was 100 μL/hr.

Conflicts of Interest

The authors declare no conflict of interest.

Contributions

Z.T. performed all experiments and simulations. Z.T and T.P.J.K. conceived and designed the experimental setup. Z.T. and T.P.J.K. wrote the main manuscript text and Z.T. prepared the figures. All authors reviewed the manuscript.
